# Deep learning for pulmonary embolism detection on computed tomography pulmonary angiogram: a systematic review and meta-analysis

**DOI:** 10.1038/s41598-021-95249-3

**Published:** 2021-08-04

**Authors:** Shelly Soffer, Eyal Klang, Orit Shimon, Yiftach Barash, Noa Cahan, Hayit Greenspana, Eli Konen

**Affiliations:** 1 Internal Medicine B, Assuta Medical Center, Samson Assuta Ashdod University Hospital, Ashdod, Israel; 2 Ben-Gurion University of the Negev, Be’er Sheva, Israel; 3 Deep Vision Lab, The Chaim Sheba Medical Center, Ramat Gan, Israel; 4 Department of Diagnostic Imaging, Sheba Medical Center, Tel Hashomer, Israel; 5 Sackler Medical School, Tel Aviv University, Tel Aviv, Israel; 6 Department of Population Health Science and Policy, Institute for Healthcare Delivery Science, Mount Sinai, New York, NY USA; 7 Sheba Talpiot Medical Leadership Program, Tel Hashomer, Israel; 8 Department of Anesthesia, Rabin Medical Center, Beilinson Hospital, Petah Tikva, Israel; 9 Department of Biomedical Engineering, Faculty of Engineering, Tel-Aviv University, Tel Aviv, Israel

**Keywords:** Medical research, Outcomes research, Cardiovascular diseases

## Abstract

Computed tomographic pulmonary angiography (CTPA) is the gold standard for pulmonary embolism (PE) diagnosis. However, this diagnosis is susceptible to misdiagnosis. In this study, we aimed to perform a systematic review of current literature applying deep learning for the diagnosis of PE on CTPA. MEDLINE/PUBMED were searched for studies that reported on the accuracy of deep learning algorithms for PE on CTPA. The risk of bias was evaluated using the QUADAS-2 tool. Pooled sensitivity and specificity were calculated. Summary receiver operating characteristic curves were plotted. Seven studies met our inclusion criteria. A total of 36,847 CTPA studies were analyzed. All studies were retrospective. Five studies provided enough data to calculate summary estimates. The pooled sensitivity and specificity for PE detection were 0.88 (95% CI 0.803–0.927) and 0.86 (95% CI 0.756–0.924), respectively. Most studies had a high risk of bias. Our study suggests that deep learning models can detect PE on CTPA with satisfactory sensitivity and an acceptable number of false positive cases. Yet, these are only preliminary retrospective works, indicating the need for future research to determine the clinical impact of automated PE detection on patient care. Deep learning models are gradually being implemented in hospital systems, and it is important to understand the strengths and limitations of these algorithms.

## Introduction

Pulmonary embolism (PE) is associated with significant morbidity and mortality^1,2^. Prompt and accurate diagnosis allows for expediting treatment. This is critical as it could substantially reduce mortality and improve outcomes^3^.

Computed tomographic pulmonary angiography (CTPA) has become the gold standard diagnostic modality for PE^4–6^. CTPA is a non-invasive, widely available, and rapidly acquired modality. However, the diagnosis of PE in CTPA is time-consuming and requires radiologists’ expertise. As a result, the interpretation process is susceptible to errors and delayed diagnosis^7,8^.

In the past few years, artificial intelligence (AI) has made a significant impact on healthcare. Specifically, deep learning algorithms, which excel at pattern recognition, are revolutionizing medical imaging analysis^9,10^.

Deep learning technology presents an innovative approach to PE detection. In this review, we present a short description of AI fundamentals followed by a literature review evaluating studies that analyzed deep learning algorithms for PE on CTPA.

## Fundamentals of artificial intelligence

### Deep learning

**Figure 1:**
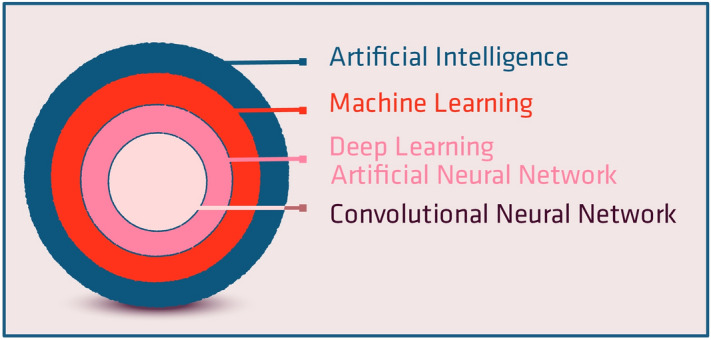
Artificial intelligence (AI) is an umbrella of terms encompassing machine learning and deep learning.

**Figure 2:**
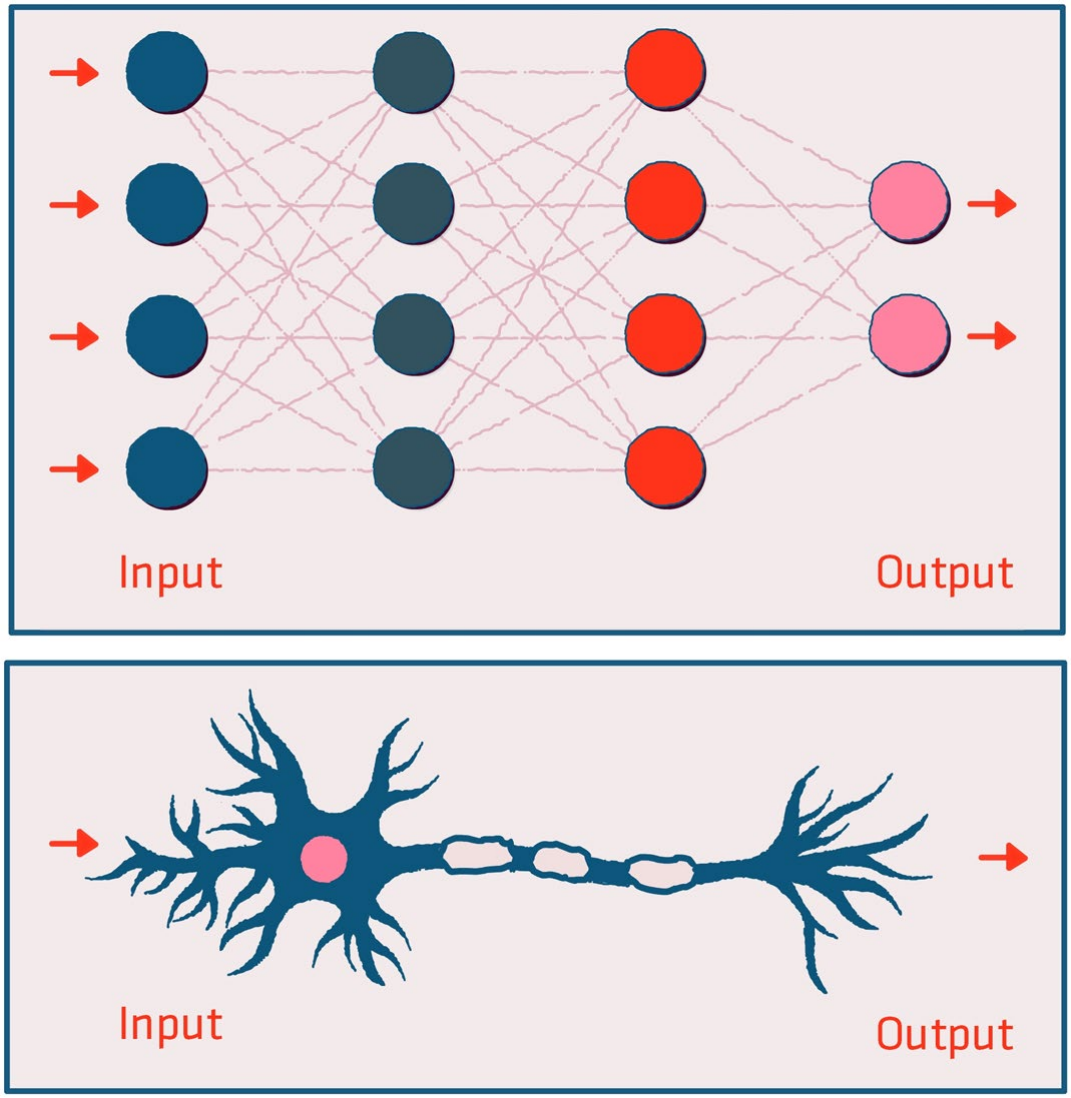
Comparison between artificial and biologic neural networks. Neural networks are comprised of multiple interconnected layers. Data is fed to the network, and an output is produced. By comparing the network’s output to the desired true label, an error can be estimated. Based on the error, the algorithm optimizes connections between the layers. The connections between the neurons are termed “weights”. Ultimately, a tuned network is achieved.

AI is a broad term that encompasses a variety of techniques (Fig. 1)^11^. Deep learning is a subfield of AI which is based on neural networks (Fig. 2). These artificial networks are composed of multiple interconnecting neuron layers. Each neuron is essentially a single linear regression unit. The inputs for each neuron are the outputs of the neurons in the previous layer. The connections between the neurons are termed “weights”.

During training, input data is fed into the network, and the final output is calculated. The difference between the network output (the estimated label) and the true label allows for error estimation. By estimating the error of the model output, the algorithm can optimize the network by tweaking its weights. This process of network optimization is called backpropagation. By tweaking the weights, important network connections are reinforced, while unimportant connections are inhibited. In this way, the difference between the network outputs and the true labels is minimized and the network's error decreases^12,13^.

### Convolutional neural networks

Convolutional neural networks (CNN) are the hallmark deep learning networks for image analysis. This algorithm was invented in the 90’ but made a major impact on the world in the 2012 ImageNet challenge^14^. That work, termed “AlexNet”, is now the most ever cited scientific paper^15^.

**Figure 3:**
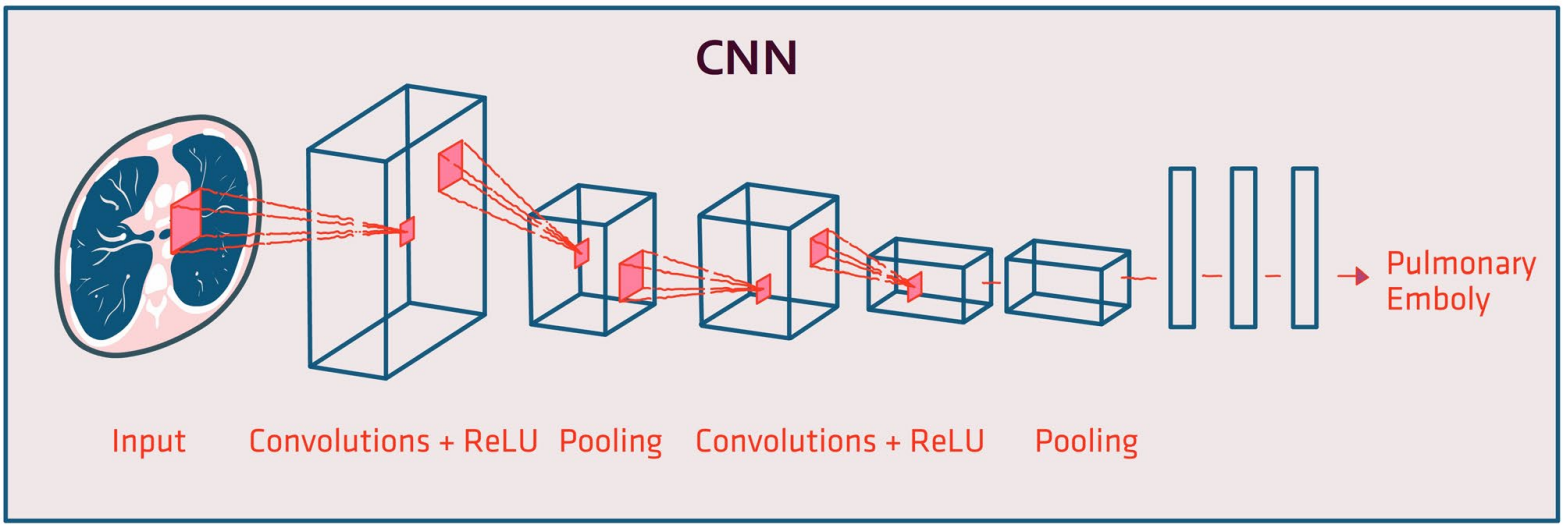
The architecture of Convolutional Neural Network (CNN). CNNs are networks specifically designed to process images. Many small filters compose each CNN layer. A filter is a small matrix of weights that is repeatedly applied to the image pixels. By sharing the filter across the image, repeating patterns are recognized. CNNs are ideal for image analysis since images are composed of repeating patterns. The shallow layers of the CNN recognize low-level patterns. The deeper layers gain a high-level understanding of the image.

CNNs are specifically designed to process images. Each CNN layer contains many filters. Each filter is a small matrix of weights, similar to the general neural networks’ weights. The filters are repeatedly applied to image pixels. Since the filters are shared across the image, they recognize repeating patterns. Thus, CNNs are ideal for image analysis, as images are composed of repeating patterns. The shallow layers of the CNN recognize low-level patterns including lines, circles, and other simple geometric patterns. The deeper layers gain a high-level understanding of the image such as context (i.e., “image with PE” vs. “image without PE”) (Fig. 3). In the past few years, CNNs made a dramatic change to medical image analysis^16^.

### Computer vision

**Figure 4:**
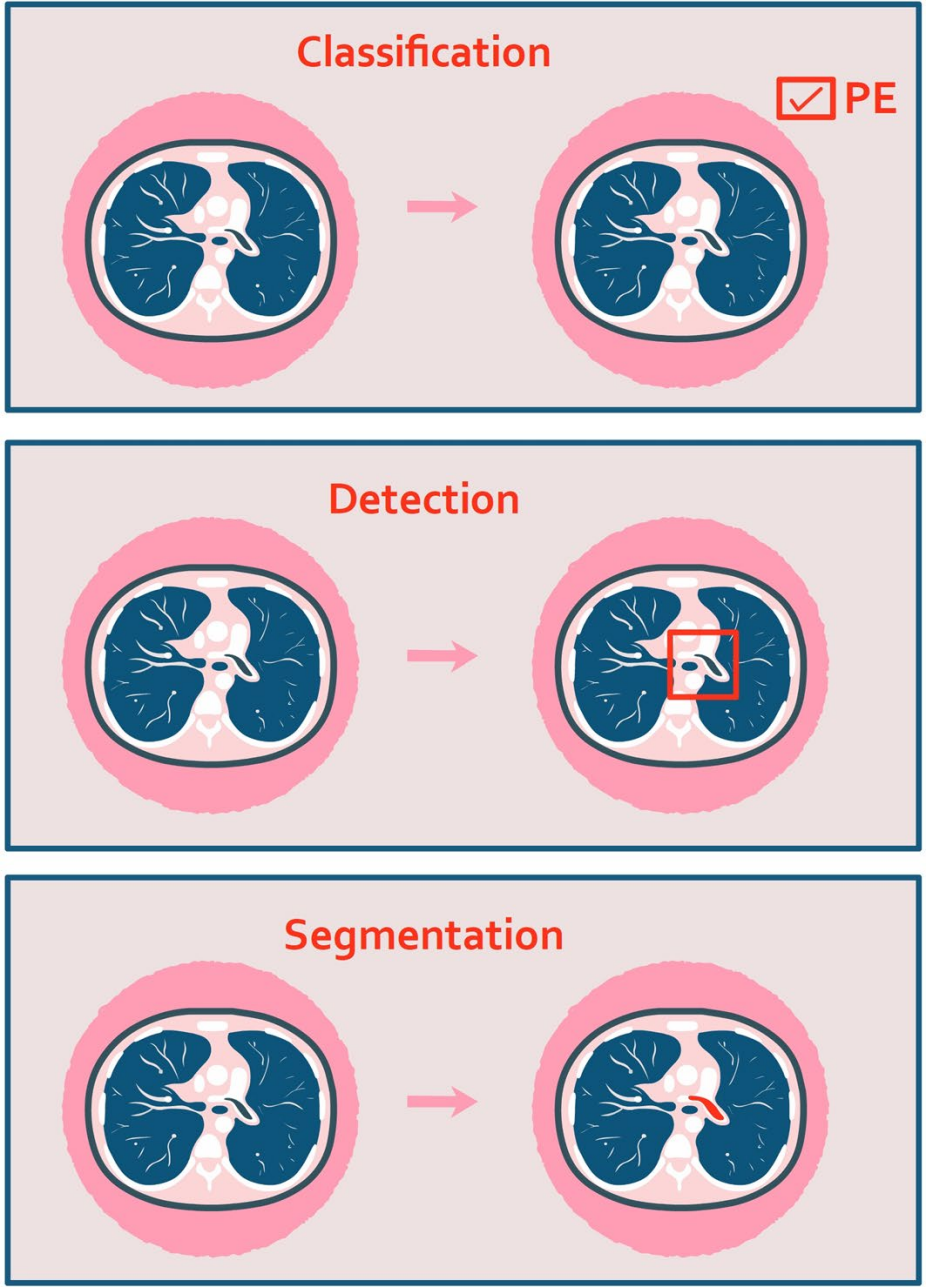
Main computer vision tasks: classification, detection, and segmentation.

Computer vision is an engineering field dedicated for analyzing images by using computer algorithms such as CNN. Three main computer vision tasks include: classification, detection, and segmentation (Fig. 4)^9^. Classification is the labeling of an entire image. Detection is the localization of an individual object in the image. Segmentation is pixel-wise delineation of the borders of an individual object in the image.

These three tasks can be understood through the analysis of CTPA with PE. The entire scan can be classified as either pathologic (with PE) or normal (no PE). We can further detect individual emboli. Lastly, we can segment the pixel-wise borders of the emboli (Fig. 4).

## Methods

This review was conducted according to the Preferred Reporting Items for Systematic Reviews and Meta-Analyses (PRISMA) guidelines^17^.

### Search strategy

A comprehensive literature search was performed to identify studies evaluating the role of deep learning in detecting PE on CTPE. The search was conducted on February 20, 2021, using the MEDLINE/PubMed databases. Search keywords included “pulmonary embolism” and “deep learning”. Details on complete search strategies are provided in Supplementary Material 1.

Inclusion criteria were studies that (1) evaluated a deep learning model for PE detection on CTPA, (2) were published in English, (3) were peer-reviewed original publications (4) and contained an outcome measure. We excluded non-computer vision articles, non-deep learning articles, and non-original articles. Abstracts were also excluded. Our search was supplemented by a manual search of references of included studies. The study is registered with PROSPERO (CRD42021237369).

### Study selection

Two reviewer authors (SS and EK) independently screened the titles and abstracts to determine whether the studies met the inclusion criteria. The full-text article was reviewed when the title met the inclusion criteria or when there was any uncertainty. Disagreements were adjudicated by a third reviewer (YB).

### Data extraction

Using a standardized data extraction sheet, the two reviewers (SS and EK) extracted data independently. Data included publication year, study design and location, number of patients, ethical statements, inclusion and exclusion criteria, description of the study population, use of an online database, size of the database, use of an independent test dataset, whether cross-validation was performed, evaluation metrics, and performance results.

### Quality assessment and risk of bias

Quality was assessed by the adapted version of the Quality Assessment of Diagnostic Accuracy Studies (QUADAS-2) criteria^18^. The studies were also evaluated using the modified Joanna Briggs Institute (JBI) Critical Appraisal checklist for analytical cross-sectional studies^19,20^.

### Data synthesis and analysis

For the quantitative meta-analysis, we used the R Statistics package mada^21^, meta, and metaprop^22^. We listed the number of true positive, true negative, false positive, and false negative results per study. Thereafter, we calculated the pooled sensitivity, specificity, and the corresponding 95% CI using the random effect model. A coupled forest plot of sensitivity and specificity was created using RevMan (version 5.3). Summary receiver operating characteristic (ROC) curves were calculated by the bivariate model of Reitsma et al.^23^. Heterogeneity was visually checked and evaluated by using I^2^. Values of I^2^ > 50% were considered as significant heterogeneity^24^.

## Results

### Study selection and characteristics

**Figure 5:**
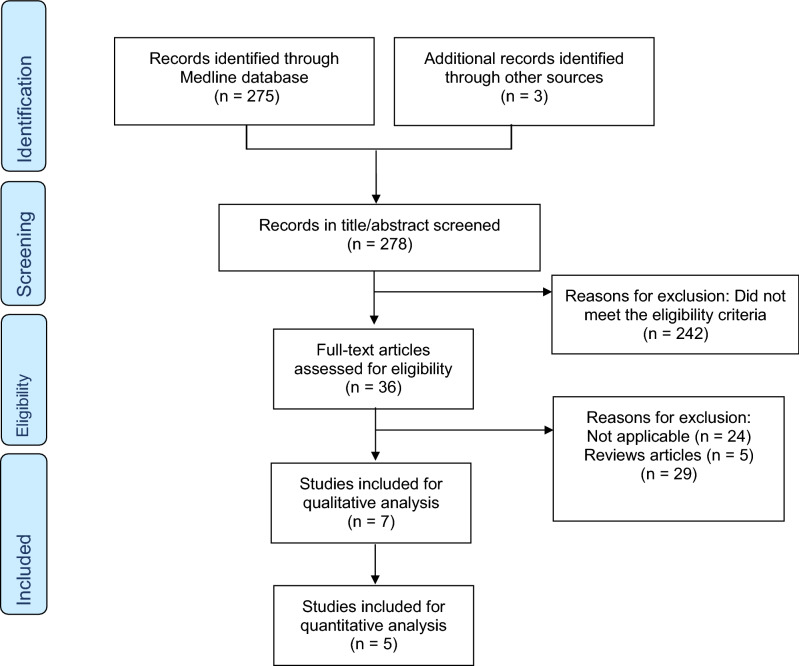
Flow diagram of the search and inclusion process.

The initial literature search resulted in 275 articles. Seven studies met our inclusion criteria (Fig. 5). Studies were published between 2015 and 2020. A total of 36,847 radiographic images were analyzed. Table 1 summarizes the characteristics of the included studies. All the studies were retrospective. In the majority of the studies (n = 6, 86%), a board-certified radiologist, served as reference standard.

**Table 1:** A summary of the articles in the literature review that applied deep learning techniques for pulmonary embolism detection on computed tomographic pulmonary angiography.

Author	Year	Study design	Database type	Dataset size (n = studies)	Images evaluated by	Performance scores
Huang et al.^27^	2020	Retrospective	Proprietary	1997	Board-certified radiologist	AUROC of 0.85 Sensitivity and specificity of 75% and 81%
Liu et al.^29^	2020	Retrospective	Proprietary	878	Delineated by two residents reviewed by an experienced chest radiologist	AUC of 0.93 Sensitivity and specificity of 94.6% and 76.5%
Huang et al.^28^	2020	Retrospective	Proprietary	1837	Board-certified radiologist	AUROC of 0.95 Sensitivity and specificity of 87.3% and 90.2%
Weikert et al.^30^	2019	Retrospective	Proprietary	29,465	Board-certified radiologist	Sensitivity and specificity of 92.7% and 95.5%
Yang et al.^40^	2019	Retrospective	Proprietary + PE challenge data	129	Board-certified radiologist	Sensitivity of 75.4% at two false positives per volume
Rajan et al. (IBM)^41^	2019	Retrospective	Proprietary	2420	Board-certified radiologists	AUC of 0.94
Tajbakhsh et al.^26^	2019	Retrospective	Proprietary + PE challenge data	121	N/A	Sensitivity of 83% at two false positives per volume

### Descriptive summary of results

Tajbakhsh et al. were the first to apply a CNN solution to detect PE^25,26^. Using 121 CTPA with 326 individual emboli, they achieved a sensitivity of 83% for detecting individual emboli at two false positives per scan. They have shown that a CNN-based solution outperforms classic machine learning techniques.

Huang et al. utilized a 3D CNN model to detect PE. They used the entire volumetric CTPA imaging data of 1971 patients and achieved an AUROC of 0.85^27^. Subsequently, they improved their model by integrating imaging data and clinical data from the electronic health record^28^. The multimodality model showed an AUROC of 0.95, outperforming single modality models.

Liu et al. deployed CNN to detect and calculate the clot burden of PE on CTPA^29^. Using 878 CTPA with 646 PE, they have shown a sensitivity of 94.6% and a specificity of 76.5%. Additionally, they displayed that the automatic measurement of clot burden was highly correlated with traditional burden scores (Qanadli and Mastora scores).

Weikert et al. developed a CNN algorithm with a relatively large training dataset consisting of 28,000 CTPAs^30^. They achieved a sensitivity of 92.7% and a specificity of 95.5%. The authors have also performed a sub-analysis which revealed that exams containing central emboli had the highest detection rates with 95.7%, followed by segmental emboli with 93.3%. Sub-segmentally located emboli had the lowest detection rate with 85.7%.

### Quality assessment

According to the QUADAS-2 tool, five papers scored as high risk of bias in at least one category. Patient selection bias was evident in more than half of the papers, as most studies failed to describe their study population. Most papers also failed in data management as ethical approval was not specified. The objective assessment of the risk of bias is reported in Supplementary Table 1 and Table 2.

### Meta-analysis results

**Figure 6:**
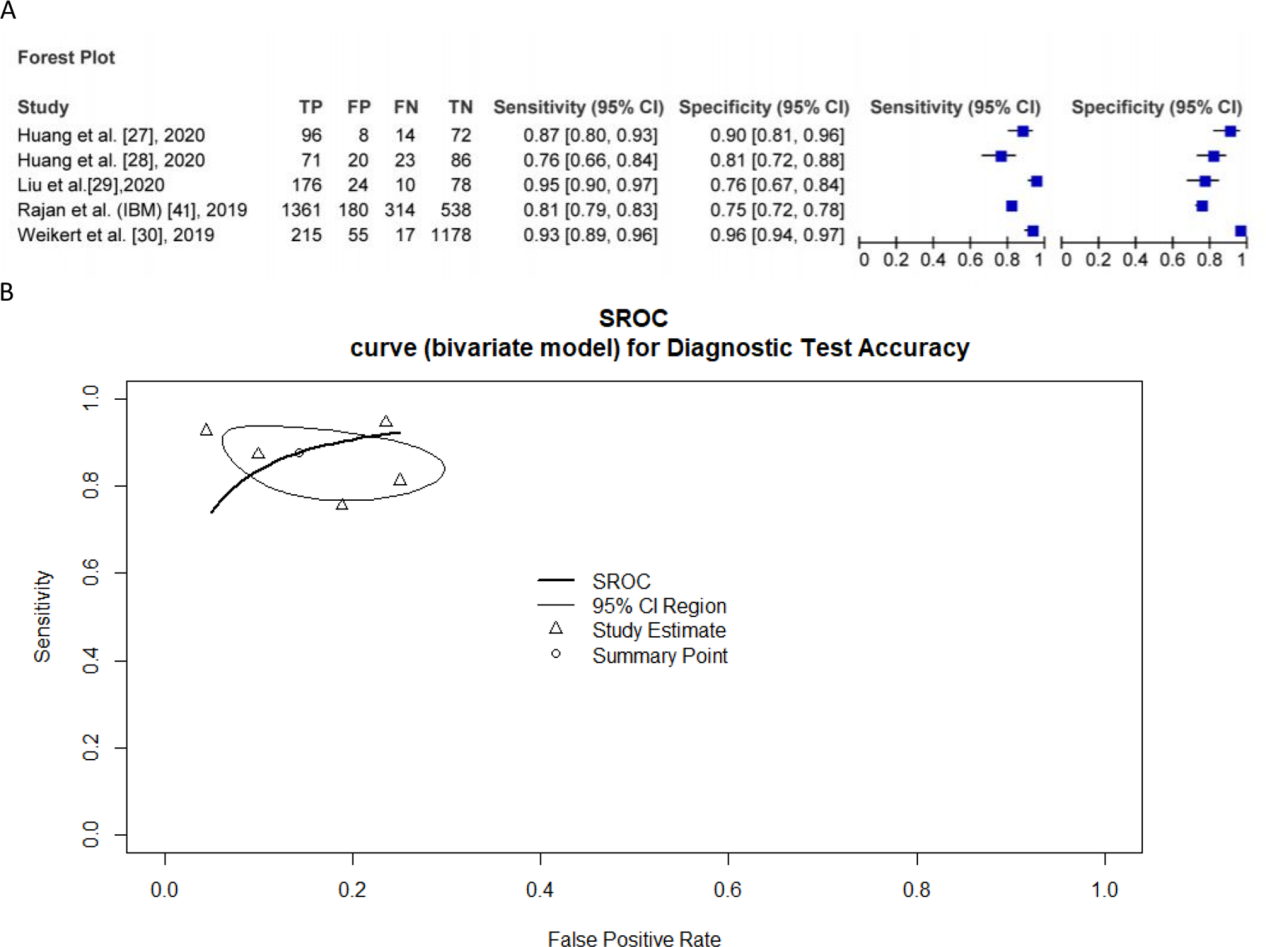
(**A**) Sensitivity and Specificity of included studies (**B**) Bivariate summary ROC curves for the detection of pulmonary embolism on CTPA using deep learning.

Five studies provided enough data to calculate test accuracies. A pooled sensitivity of 0.88 (95% CI 0.803–0.927, I^2^ = 89.6%) per scan and a specificity of 0.86 (95% CI 0.756–0.924, I^2^ = 97.4%) per scan were shown. Figure 6 presents the sensitivity, specificity, and the bivariate summary ROC curve.

## Discussion

Accurate and rapid diagnosis of PE is essential to improve prognosis. Previous research raised the concern that radiologists’ interpretation may be impaired by a lack of sensitivity for PE detection. It was demonstrated that the radiologists’ sensitivity for detecting PE ranges from 0.67 to 0.87 with a specificity of 0.89 to 0.99^31–33^. The presented deep learning models provide an automatic approach for identifying PE on CTPA with a pooled sensitivity of 0.88 and specificity of 0.86.

An effective AI system must have an optimal operating threshold that balances between sensitivity and specificity. Such systems can accelerate the diagnostic workflow without burdening the radiologist with false positive cases as a high number of false positives creates alarm fatigue^34^. For PE detection, it is apparent that a deep learning system can serve as a second reader for the immediate interpretation and prioritization of positive studies. Ultimately, an AI-based tool has the potential to reduce the time to PE diagnosis. Since timely diagnosis is critical, the integration of a triage model can enhance the quality of care. Liu la et al. demonstrated that a deep learning model could also flag patients with a worse prognosis according to clot burden or right ventricular dysfunction parameters^29^.

Early work in automated PE diagnosis was based on traditional machine learning techniques^35–37^. Commercially available PE detection solutions based on machine learning were also developed^38–40^. Nonetheless, moderate success with a limited clinical application was achieved. These techniques were tested only on small cohorts. Additionally, even though they achieved clinically acceptable sensitivities, it was at the cost of an extremely high number of false positive cases. Indeed, existing applications were not widely utilized. Deep learning models obtained more promising results with high sensitivity at an acceptable false positive rate.

Although a significant improvement was attained with deep learning, these achievements are limited and are based on a small number of studies. Except for one research^28^, the studies did not leverage the abundant amount of tabular data on each patient, such as comorbidities and laboratory results. Moreover, all the reviewed studies were retrospective and were not tested in the clinical setting. A direct comparison between the deep learning algorithm and the radiologist performance was not carried out. Multicenter prospective studies are currently missing. It is crucial to evaluate whether an automatic PE detection system can improve the radiologist’s performance, ultimately resulting in better clinical outcomes.

In the 2020 annual meeting of the Radiological Society of North America (RSNA), a competition was conducted to detect PE in CTPA studies^41^. A large publicly available dataset that included 12,000 CT scans was created for the challenge. These scans were provided by five international medical centers and were annotated by 80 board-certified thoracic radiologists. It is expected that studies based on this public database will be published in the near future.

Several commercial companies also specialize in developing deep learning algorithms to flag and triage urgent PE on CTPA^42^. One company received FDA clearance for their AI tool^42^. In the near future, decision support systems for the detection of PE will be implemented as a second reader. Next, depending on the technology advancement, these systems are expected to replace some of the radiologist’s role. For example, in the future, the AI system may have the potential to filter the normal scans with high accuracy, thereby allowing the radiologist to focus on interpreting the abnormal and complicated cases.

Our review has several limitations. All of the reviewed studies were retrospective. The studies’ heterogeneity limited assessment of the pooled performance. Half of the studies were at high risk of bias. All studies were conducted in an experimental setting only. Additional studies will be needed to confirm the usefulness of the tool.

In conclusion, deep learning models can detect PE on CTPA with satisfactory sensitivity and an acceptable number of false positive cases. Yet, these are only preliminary retrospective works, indicating the need for future research to determine the clinical impact of automated PE detection on patient care. Deep learning models are gradually being implemented in hospital systems, and it is important to understand the strengths and limitations of these algorithms.

## Supplementary Information

Supplementary Information 1.

Supplementary Information 2.

## Data Availability

All data generated or analysed during this study are included in this published article (and its Supplementary Information files).
