# Supplementary material for: Deep learning for pulmonary embolism detection on computed tomography pulmonary angiogram: a systematic review and meta-analysis
Source: Sci Rep. 2021 Aug 4;11:15814. doi: 10.1038/s41598-021-95249-3 (PMC8338977; doi:10.1038/s41598-021-95249-3)
Supplement: Supplementary file 1 — Supplementary Information 1. [file 41598_2021_95249_MOESM1_ESM.docx]

**Supplementary Online Content**

**Supplementary Material 1.** Literature search strategy…………..……………..…..…..… 2

**Supplementary Table 1**. Quality Assessment of Diagnostic Accuracy Studies-2......…..3

**Supplementary Table 2.** The modified Joanna Briggs Institute (JBI) checklist.....…...…4

**References**………………………………………………………………………….……….…..5

This supplementary material has been provided by the authors to give readers additional information about the work.

**Supplementary Material 1: Literature search strategy**

Database: Ovid MEDLINE(R) and Epub Ahead of Print, In-Process & Other Non-Indexed Citations and Daily <1946 to February 14, 2021>

Search Strategy:

--------------------------------------------------------------------------------

1 ((PE) OR (pulmonary embolism)).mp.

2 ((((((deep learning) OR (CNN)) OR (convolutional neural networks)) OR (neural network)) OR (neural networks))).mp.

3 1 and 2

***************************

**Supplementary Table 1: Quality Assessment of Diagnostic Accuracy Studies-2 (QUADS-2) risk of bias assessment**.

Abbreviations: Pt. patient; Ref. reference.
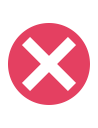
 = high risk of bias;
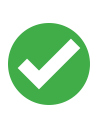
 = low risk of bias.

^a^Failing to describe their study population; ^b^external validation; ^c^Who performed the annotations? ^d^Did all patients receive the same reference standard? Were all patients included in the analysis? ^e^Failing to specify ethical approval.

|  | **Risk of bias** | | | | |
| --- | --- | --- | --- | --- | --- |
| **Author** | **Pt. selection^a^** | **Index test^b^** | **Ref. standard^c^** | **Flow and timing^d^** | **Data management^e^** |
| Huang et al. [^1^](#_ENREF_1) | 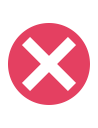 | 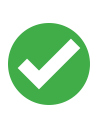 | 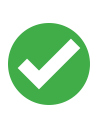 | 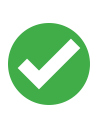 | 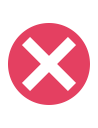 |
| Liu et al. [^2^](#_ENREF_2) | 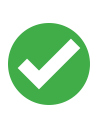 | 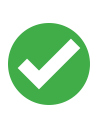 | 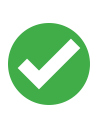 | 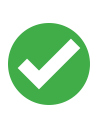 | 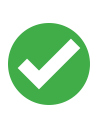 |
| Huang et al. [^3^](#_ENREF_3) | 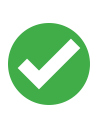 | 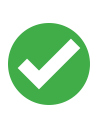 | 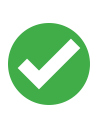 | 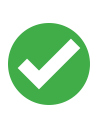 | 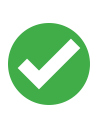 |
| Weikert et al. [^4^](#_ENREF_4) | 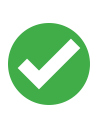 | 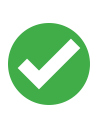 | 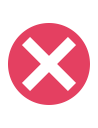 | 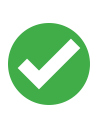 | 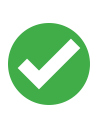 |
| Yang et al. [^5^](#_ENREF_5) | 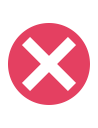 | 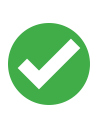 | 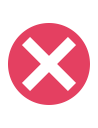 | 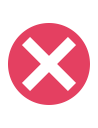 | 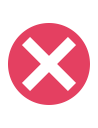 |
| Tajbakhsh et.al [^6^](#_ENREF_6) | 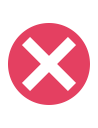 | 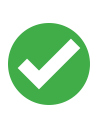 | 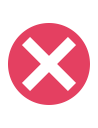 | 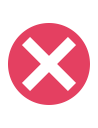 | 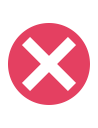 |
| Rajan et al. (IBM) [^7^](#_ENREF_7) | 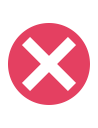 | 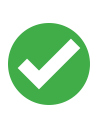 | 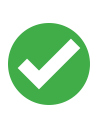 | 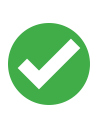 | 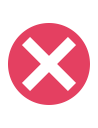 |

**Supplementary Table 2: The modified Joanna Briggs Institute (JBI) critical appraisal checklist for cross-sectional research.**

| **Item** | **Checklist** | **Applicability** |
| --- | --- | --- |
| **1** | Were the objective of the study and the criteria for inclusion in the sample (data selection) clearly defined? | **42%** |
| **2** | Were the study subjects and the setting described in detail? Were the sample population and variables described in details? (i.e. how were the images collected? Which hospital, what technique was used?) | **28%** |
| **3** | Did the study detail data source (e.g. hospital, database, survey) and format? | **42%** |
| **4** | Was the data collected in a valid and reliable way? | **42%** |
| **5** | Attainment of ethical approval. Was the ethical issue (patient confidentiality) considered? | **57%** |
| **6** | Were findings and implications discussed in detail? | **85%** |
| **7** | Were the outcomes (performance and result of ML tools) measured in a valid and reliable way? | **100%** |
| **8** | Was appropriate cross-validation and evaluation method used? | **100%** |

References:

1. Huang SC, Kothari T, Banerjee I, et al. PENet—a scalable deep-learning model for automated diagnosis of pulmonary embolism using volumetric CT imaging. *npj Digital Medicine.* 2020;3(1).

2. Liu W, Liu M, Guo X, et al. Evaluation of acute pulmonary embolism and clot burden on CtpA with deep learning. *European radiology.* 2020:1-9.

3. Huang S-C, Pareek A, Zamanian R, Banerjee I, Lungren MP. Multimodal fusion with deep neural networks for leveraging CT imaging and electronic health record: a case-study in pulmonary embolism detection. *Scientific reports.* 2020;10(1):1-9.

4. Weikert T, Winkel DJ, Bremerich J, et al. Automated detection of pulmonary embolism in CT pulmonary angiograms using an AI-powered algorithm. *European radiology.* 2020;30(12):6545-6553.

5. Yang X, Lin Y, Su J, et al. A two-stage convolutional neural network for pulmonary embolism detection from CTPA images. *IEEE access : practical innovations, open solutions.* 2019;7:84849-84857.

6. Tajbakhsh N, Shin JY, Gotway MB, Liang J. Computer-aided detection and visualization of pulmonary embolism using a novel, compact, and discriminative image representation. *Medical Image Analysis.* 2019;58.

7. Rajan D, Beymer D, Abedin S, Dehghan E. Pi-PE: A pipeline for pulmonary embolism detection using sparsely annotated 3D CT images. *arXiv preprint arXiv:1910.02175.* 2019.
